# Bone remodeling of the proximal tibia after uncemented total knee arthroplasty: secondary endpoints analyzed from a randomized trial comparing monoblock and modular tibia trays—2 year follow-up of 53 cases

**DOI:** 10.1080/17453674.2019.1637178

**Published:** 2019-07-04

**Authors:** Mikkel Rathsach Andersen, Nikolaj Winther, Thomas Lind, Henrik M Schrøder, Michael Mørk Petersen

**Affiliations:** a Department of Orthopedics, Rigshospitalet, University of Copenhagen, Copenhagen;; b Department of Orthopedics, Herlev Gentofte Hospital, Copenhagen, Denmark

## Abstract

Background and purpose — Bone remodeling as a response to bone trauma, postoperative immobilization, and device-related bone reactions can lead to loss of bone stock and increase the risk of periprosthetic fracture and aseptic loosening. This study investigates the adaptive bone remodeling of the proximal tibia after uncemented total knee arthroplasty (TKA).

Patients and methods — We performed a 2-year follow up of 53 patients (mean age 62 (38–70) years, 27 of whom were men, who received an uncemented TKA in a randomized controlled trial with bone mineral density (BMD) as secondary endpoint. Patients were randomized to 2 groups of either monoblock (A) or modular (B) polyethylene design. The TKAs were performed using the uncemented Zimmer Nexgen trabecular metal. Measurements of BMD were done postoperatively and after 3, 6, 12, and 24 months. BMD was measured in 3 regions of interest (ROI).

Results and interpretation — In group A statistically significant changes in BMD were seen after 24 months in both the medial and lateral ROI. BMD decreased medially by 15% (p = 0.004) and laterally by 13% (p = 0.01). In group B the BMD changes were limited and after 24 months returned to the preoperative values. The differences in BMD change between groups were statistically significant in both the medial (p = 0.03) and lateral (p = 0.02) ROI. In the distal ROI we found no significant change in BMD in either group. A significantly different bone remodeling pattern of the proximal tibia was seen in the 2 groups with a higher degree of bone loss in the knees that received the monoblock polyethylene design, indicating that the flexible monoblock implant design, previously shown to improve fixation, does not decrease the bone loss of the proximal tibia.

Bone remodeling after joint replacement surgery is a well-known consequence of bone trauma, immobilization, and redistribution of the mechanical loading after joint arthroplasty surgery (Bohr and Lund 1987, Järvinen and Kannus 1997, Soininvaara et al. 2004b, Andersen et al. 2018). After total knee arthroplasty (TKA) bone remodeling occurs in both the proximal tibia and distal femur. Most studies have found TKA to result in a decrease in bone mineral density (BMD) of the tibia and femur (Bohr and Lund 1987, Levitz et al. 1995, Petersen et al. 1995, 1996b, Soininvaara et al. 2004c).

The long-term fixation of uncemented tibial components relies on bone ingrowth. The decrease in BMD of the bone is clinically important as BMD is directly related to the breaking strength of the bone (Hansson et al. 1980, Hvid et al. 1985, Petersen et al. 1996a), hence increasing the risk for complications such as periprosthetic fractures; also, the bone loss complicates revisions of the tibial components, and most importantly is related to a high degree of migration and possibly aseptic loosening (Andersen et al. 2017).

In this study we investigated the bone remodeling of the proximal tibia after implantation of the Trabecular Metal Technology (TMT) Zimmer Nexgen (Zimmer Biomet, Warsaw, IN, USA), which has a porous tantalum surface. Porous implant surfaces enhance bone ingrowth at the bone–implant interface (Bobyn et al. 1980, 1999, Engh et al. 1987, 1995).

In the monoblock implant design the polyethylene is compression-molded directly onto the trabecular metal back. This design in theory eliminates backside wear of the polyethylene.

Besides eliminating backside wear the monoblock design also gives the component mechanical properties very similar to those of cancellous bone in terms of low stiffness and high resistance to compressive stress. The modular tibial component is designed with a titanium plate molded on top of the tantalum trabecular metal in order to create a locking mechanism for the polyethylene (Andersen et al. 2016). This allows the surgeon to change only the polyethylene but can lead to backside wear of the polyethylene. Molding a titanium plate on top of the trabecular metal back, however, makes the modular component stiffer than the monoblock component. These differences in mechanical properties and polyethylene wear could affect the bone remodeling of the tibia (Engh et al. 1987, Bobyn et al. 1999).

In this this study we quantify the adaptive bone remodeling of the proximal tibia after uncemented TKA using dual X-ray absorptiometry (DEXA) in a prospective randomized setting comparing monoblock versus modular tibia component design. The bone remodeling of the tibia was a secondary endpoint in a previously published RSA study designed to investigate the migration of uncemented tibia components (Andersen et al. 2016). The randomized RSA study found lower migration of the monoblock group compared with the modular group; however, both implants followed the expected migration pattern of cementless implants, that is high initial migration followed by stabilization from 6 to 24 months postoperatively.

## Patients and methods

### Patients and implants

75 patients scheduled for TKA surgery with a cruciate-retaining TKA because of osteoarthritis were included in a prospective randomized clinical trial with 2 years of follow-up. All patients included were under 70 years of age at the date of the operation, and suffered no bone-related diseases other than osteoarthritis. The patients were randomized to receive the monoblock or modular polyethylene design version of the Cruciate Retaining Trabecular Metal Technology Nexgen tibial component (Andersen et al. 2016). The femur components used in all patients were the cruciate-retaining, uncemented titanium Zimmer Nexgen Flex, and all patients had a cemented Nexgen all poly patella component.

The cohort of 53 patients, 26 with monoblock, 27 with modular, included and followed in this study are identical to those of the RSA study previously published and a flowchart and demographics table are included in that publication (Andersen et al. 2016). The 2 groups of patients were comparable in all preoperative demographics, and substantial and similar improvements in clinical results were seen in both groups.

### DEXA scans

The DEXA scans were done using a Norland XR-46 bone densitometer (Norland Corp., Fort Atkinson, WI, USA). We used customized software with an adjustable threshold for metal exclusion allowing BMD measurements of the bone adjacent to the implants. Scan speed was set at 45 mm/sec, and a pixel size of 0.5 Ч 0.5 mm. The proximal tibias were scanned in the coronal plane with patients in a standardized supine position, with the knee extended and lower limb slightly internally rotated to avoid tibia–fibula overlay. We also measured BMD of the distal tibia and fibula 1 cm above the ankle joint in all patients in the operated and non-operated limbs. All DEXA scans were performed by an experienced laboratory technician. Patients were scanned postoperatively within 1 week and with follow-ups after 3, 6, 12, and 24 months.

**Figure 1.**
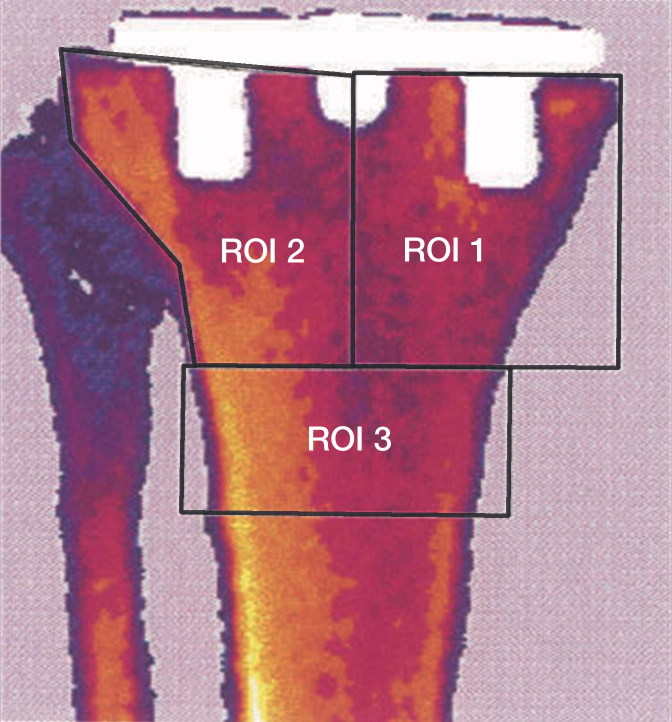
DEXA scan of the tibia in the anterior–posterior projection with the three regions of interest. ROI 1: Periprosthetic tibial bone from the center of prosthesis to medial edge of the proximal tibia, length 4 cm. ROI 2: Periprosthetic tibial bone from the center of the prosthesis to the lateral edge of the tibia, length 4 cm. ROI 3: Full width of the tibia distal to ROI 1 and RO 2, length 2 cm.

On the computerized scan images, we created 3 regions of interest (ROI) in which we measured the periprosthetic BMD changes over time (Figure 1). ROI 1 is a 4 cm long region ranging in width from the medial side of the tibia to the center of the tibia component. ROI 2 is a 4 cm long region stretching from the lateral side of the tibia to the center of the tibia component. ROI 3 is a 2 cm long region located distally to ROI 1 and ROI 2 stretching the entire width of the tibia (Figure 1).

DEXA scans were also performed at the ankles, where we created ROI 4 located 1 cm above the ankle joint including both the tibia and the fibula with a length of 2 cm. The ankle bone mineral is reported as bone mineral content (BMC) in grams. We chose BMC because it is more accurate and reproducible than measuring BMD at the ankles due to the patient variation in tibia–fibula overlay (Figure 1). This problem is not relevant in the proximal tibia measurements.

Precision error of the tibia BMD measurements was calculated by double DEXA scans of 23 knees and expressed as the mean coefficient of variation (CV). Patients were asked to step off the DEXA scanner, wait for 5 minutes and were then replaced in the standardized supine position and the DEXA scan repeated.

### Statistics

The minimal relevant difference in BMD changes for this study was set at 7.5%. A previous study of BMD changes in the proximal tibia after uncemented TKA has found an SD of 7.5% (Petersen et al. 1995). Calculating the sample size (type I error = 5% and type II error = 15%) we found a sample size of 20 in each group. Since the patients included in this study also participated in an RSA study (Andersen et al. 2016) requiring 30 patients per group, we also included 30 patients per group for this study, leaving room for dropouts during the 2-year follow-up period.

The BMD data in both groups could be considered normally distributed. We used the unpaired t-test to test for differences in BMD changes over time between the groups after 24 months, and repeated measures ANOVA for BMD change over time within the groups. Statistical analyses were done using the SPSS 21.0 software (IBM Corp., Armonk NY, USA). P-values below 0.05 were considered significant.

### Ethics, registration, funding, and potential conflicts of interest

The study was approved by the Scientific Ethical Committee of Copenhagen (H-1-2012-033), and conducted in accordance with the Helsinki declaration with informed consent obtained (after written and oral information) from all study participants prior to inclusion in the study. The manuscript was written in accordance with the CONSORT guidelines for randomized trials. The study was approved by the Danish Data Protection Agency (ID 01766, GEH-2012-027), and the study was registered at ClinicalTrials.gov (NCT01637051) prior to study start.

The study received financial support Zimmer Inc. (Warsaw, IN, USA) and from Gentofte Hospital (research grant). No competing interests were declared.

## Results

A table with the clinical outcome scores, and preoperative parameters including preoperative knee alignment, has previously been published (Andersen et al. 2016).

The precision expressed as mean (SD) CV of the tibia BMD measurements was 2.9% (2.5%), 2.1% (1.9), and 0.2% (0.2) for respectively ROI 1, ROI 2, and ROI 3.

In the medial region (ROI 1) we found an increase in BMD in the modular group of 3.7% during the first 3 months, whereas in the monoblock group BMD decreased by 1.3%. After the first 3 months the BMD in ROI 1 of both groups decreased and did so for the rest of the 24 months’ follow-up period. In the monoblock group the BMD decreased by 4.7%, 9.4%, and 15% in ROI 1 after 6, 12, and 24 months, respectively (Table, see Supplementary data, Figure 2). In the modular group the BMD decreased slowly from 3 to 24 months, when it was close to the postoperative level. When comparing the changes in BMD between the two groups in ROI 1 during the 24 months of follow-up, we found a statistically significant larger BMD decrease in the monoblock group at 24 months of follow-up (p = 0.03).

**Figure 2.**
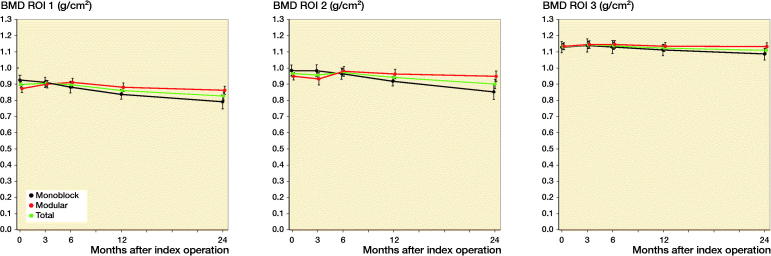
Change in bone mineral density (BMD [SE]) in ROI 1, ROI 2, and ROI 3 of the proximal tibia.

In the lateral region (ROI 2) we also found a BMD decrease in the monoblock group over the 24 months’ follow-up period. After 24 months, the BMD of the monoblock group had decreased to 13% below the postoperative value, while in the modular group only limited insignificant changes were seen at 24 months of follow-up, where BMD was almost on the same level as immediately after the operation. However, as in ROI 1, we found a 6-month increase in the modular group BMD of ROI 2, followed by a slow decrease towards 24 months. When comparing the changes in BMD between the groups in ROI 2 at the 24 months’ follow-up, we found a statistically significant (p = 0.02) difference.

In the distal region (ROI 3) the BMD changes in the 2 groups were very limited and statistically insignificant. Furthermore, we found no significant differences between the 2 study groups.

The BMC of the ankles was measured in both the operated and non-operated limbs. In general, we found only small non-significant changes in BMC of the ankles. There was no significant difference between the two groups regarding BMD changes of the ankle at any time during the follow-up.

## Discussion

To our knowledge, there are no previous studies of bone remodeling of the proximal tibia after uncemented TKA with the modular trabecular metal tibia component. Also, there are no previous studies of tibia bone remodeling comparing the modular versus monoblock design in uncemented TKA.

A limitation of this study is that the bone mineral data were collected as a secondary endpoint to the primary RSA study. The study is therefore considered exploratory, investigating whether the difference in mechanical properties and polyethylene wear of the monoblock and modular tibia tray designs has an effect on the bone remodeling of the proximal tibia.

The BMD change in our study was not equally distributed in the 2 groups. The monoblock group decreased 15% in ROI 1, 13% in ROI 2, and 3.5% in ROI 3. In contrast, the BMD of the modular group was almost identical to the postoperative value after 2 years.

Previous studies of bone remodeling of the proximal tibia after TKA with uncemented tibial components have found quite varying results. Petersen et al. (1995) found a decrease of 22% after 3 years. Levitz et al. (1995) found a 5% decrease per year (36% at 8 years), and Regnér et al. (1999) 26% at 5 years. Conversely Petersen et al. (1996a) found an increase of 6.1% in the lateral tibia after two years. Winther et al. (2016) also reported a small 24 months’ increase in BMD after uncemented TKA in the periprosthetic tibia-bone regions of 1.3%–5.5% in 57 subjects.

Minoda et al. (2013) performed a study using the same uncemented monoblock component as in the present study and compared it with a cemented tibia implant. They defined ROIs that were similar to ours. They found BMD changes of –41%, –12%, and –4% in the regions corresponding to our ROI 1, ROI 2, and ROI 3 respectively after 5 years. To our knowledge there are no previous bone remodeling studies of the modular component.

The BMD of proximal ROI1 and RIO2 of the modular group increased during the first 6 months. A possible explanation for this could be the result of a larger average realignment correction in the modular group. Tables presenting preoperative demographics and clinical parameters including pre- and postoperative alignment were presented in our previous publication (Andersen et al. 2016). The altered weight transfer after realignment to a more physiologic alignment with altered mechanical loading of the proximal tibia postoperatively could explain the initial increase in ROI 1 and ROI 2. This temporary increase as an effect of realignment in TKA has previously been reported (Bohr and Lund 1987, Soininvaara et al. 2004b). Patients with knee arthrosis are known to have a higher BMD of the proximal tibia than in healthy knees and part of the postoperative bone loss could represent a return to normal (Madsen et al. 1994). This could also explain part of the difference between our groups as the postoperative BMD of ROI 1 of the monoblock group was 8% above the modular group (0.93 g/cm^2^ versus 0.87 g/cm^2^) and 5% above the modular group in ROI 2 (0.98 g/cm^2^ versus 0.95 g/cm^2^). In ROI 3 the 2 groups had identical average postoperative BMD values of 1.3 g/cm^2^. The result that stands out most when comparing our results with previously reported results is the lack of bone loss in the modular group during the 2-year follow-up. We believe that the best explanation for this is the above-mentioned lower average starting point BMD and the larger shift in mechanical loading due to realignment, rather than mechanical properties specific to the modular design; if the follow-up period had been longer, the BMD graphs for the modular group probably would continue to decline at a rate similar to that of the monoblock group.

The difference in BMD change between the groups did not reflect different knee function of the subjects, and our previous publication (Andersen et al. 2016) documented a good clinical outcome in both groups concerning Knee Society Scores and life quality and we found no statistically significant difference between the groups for these parameters.

We also performed BMD measurements of the distal tibia at the ankles in all patients to ensure that the BMD change in the proximal tibia was a true local response to the operation and tibial component implantation and not an overall decrease in skeletal BMD or a result of immobilization of the operated limb. The BMD changes of the ankles were statistically insignificant and smaller than BMD changes of the proximal tibia, indicating that the significant BMD change of the ROIs in the proximal tibia was largely a true local response to the TKA surgery. However, they tended to follow the same pattern with a decrease in BMD in the monoblock group of both the operated and non-operated limbs, whereas in the modular group the BMD increased during the follow-up period (data not shown).

The initial BMD increase in the modular group was followed by a slow decrease in BMD over the next 18 months with a slope similar to that of the monoblock group, indicating that a longer follow-up period would also have resulted in negative BMD changes in the modular group. The BMD of all the 3 ROIs in all subjects in this study decreased at a rate of 3.4% per year. This result corresponds relatively well to the 26% 5-year decrease in the study by Regnér et al. (1999), and the 40% 8-year decrease seen in the study by Levitz et al. (1995).

From the RSA study of these patients we knew that the flexible monoblock component migrated statistically significantly less than the rigid modular component, which led us to hypothesize that we would find less bone loss in the monoblock group. We attributed the lower migration of the monoblock component to better mechanical properties and reduced backside wear and expected the superior fixation to result in a lower degree of bone loss. However, our findings indicate that this hypothesis was false. It is likely that other parameters than the degree of implant fixation have a greater effect on bone remodeling of the tibia; an example of such factors could be weight-transfer shifts after knee realignment and differences in preoperative level of BMD. Studies of distal femur bone remodeling after TKA support this notion, as the posterior shift in weight transfer and the absence of patellar pressure after TKA are believed to explain the large difference in bone loss anteriorly and posteriorly in the distal femur (Soininvaara et al. 2004c), Andersen et al. 2018). Though we did not find the expected relation between implant design and bone remodeling we do consider investigating such relations important becauses the bone loss of the tibia is clinically important as BMD is directly related to the breaking strength of the bone and complicates revision surgery (Hansson et al. 1980, Hvid et al. 1985, Petersen et al. 1996a). We considered a minimal difference of 7.5% BMD change between the groups to be clinically significant. We conclude that we did find a clinically relevant difference in the amount of bone loss in ROI 1 and ROI 2 over the 2-year follow-up, when compared with an expected bone loss of 1–2% in the general population during a 2-year period, but the difference probably should not be attributed to the mechanical properties of the tibial implants as hypothesized. A TKA is expected to last at least a 15-year period during which the BMD of the proximal tibia could be expected to decrease by approximately 50–80% assuming the BMD loss continues over longer periods at the reported rates. Such loss of tibia bone quality could result in serious complications such as implant loosening and periprosthetic fractures, and provide surgical difficulties in TKA revisions. As TKA patients become younger, poor tibia bone quality could represent an increasing problem in future revision arthroplasty surgery.

### Supplementary data

The Table is available as supplementary data in the online version of this article, http://dx.doi.org/10.1080/17453674. 2019.1637178

MRA: Study conception and design, acquisition of data, analysis and interpretation of data, drafting of manuscript, critical revision. NW: Study conception and design. TL: Study conception and design, acquisition of data. HMS: Study conception and design, acquisition of data. MMP: Study conception and design, analysis and interpretation of data, critical revision.

The authors would like to thank Karen Elisabeth Sønderlev for technical support performing DEXA scans.

*Acta* thanks Harald Brismar and Rüdiger J Weiss for help with peer review of this study.

## Supplementary Material

Supplemental Material
