# Supplementary material for: Bone remodeling of the proximal tibia after uncemented total knee arthroplasty: secondary endpoints analyzed from a randomized trial comparing monoblock and modular tibia trays—2 year follow-up of 53 cases
Source: Acta Orthop. 2019 Jul 4;90(5):479–83. doi: 10.1080/17453674.2019.1637178 (PMC6746298; doi:10.1080/17453674.2019.1637178)
Supplement: Supplemental Material [file IORT_A_1637178_SM7846.pdf]

Supplementary data

2-year follow-up results of BMD measurements of the proximal tibia (g/cm^2), and ΔBMD (%) of the proximal tibia ROI 1, ROI 2, and ROI 3 and BMC (g) of the ankles (ROI 4).

	Postoperative		3 months		6 months		12 months		24 months	
	Monoblock	Modular	Monoblock	Modular	Monoblock	Modular	Monoblock	Modular	Monoblock	Modular
**ROI 1, BMD (g/cm^2)**										
Mean (SD)	0.93 (0.16)	0.87 (0.13)	0.91 (0.17)	0.90 (0.12)	0.88 (0.18)	0.89 (0.14)	0.84 (0.15)	0.88 (0.15)	0.79 (0.22)	0.86 (0.14)
ΔBMD (%)			–1.3	3.7	–4.7	3.2	–9.4	–0.2	–15.0	–0.01
p-value [a]									0.004	0.03
p-value [b]									0.03	
**ROI 2, BMD (g/cm^2)**										
Mean (SD)	0.98 (0.17)	0.95 (0.13)	0.98 (0.18)	0.93 (0.20)	0.96 (0.18)	0.98 (0.16)	0.92 (0.15)	0.98 (0.16)	0.85 (0.24)	0.95 (0.17)
ΔBMD (%)			0.2	–1.8	–1.9	3.1	–6.7	0.8	–13.3	0.0
p-value [a]									0.01	0.5
p-value [b]									0.02	
**ROI 3, BMD (g/cm^2)**										
Mean (SD)	1.13 (0.15)	1.13 (0.11)	1.14 (0.18)	1.15 (0.12)	1.13 (0.21)	1.14 (0.13)	1.11 (0.18)	1.13 (0.13)	1.09 (0.18)	1.13 (0.13)
ΔBMD (%)			1.0	1.3	0.1	0.8	–1.5	–0.2	–3.5	0.0
p-value [a]									0.8	0.5
p-value [b]									1.0	
**Ankle of operated limb, BMC (g)**										
Mean (SD)	7.89 (0.49)	7.77 (0.42)	7.50 (0.37)	7.97 (0.34)	7.70 (0.38)	8.14 (0.33)	7.54 (0.38)	8.21 (0.36)	7.60 (0.36)	8.19 (0.32)
ΔBMD (%)			–5.0	2.6	–2.4	4.8	–4.5	5.8	–3.7	5.4
p-value [a]									0.7	0.6
p-value [b]									0.2	
**Ankle of non-operated limb, BMC (g)**										
Mean (SD)	8.01 (0.39)	7.97 (0.40)	8.05 (0.38)	8.42 (0.30)	8.09 (0.39)	8.48 (0.35)	7.75 (0.36)	8.53 (0.34)	7.94 (0.36)	8.45 (0.34)
ΔBMC (%)			0.4	5.7	1.0	6.5	–3.2	7.1	–0.9	6.1
p-value [a]									0.9	0.4
p-value [b]									0.3	

[a] Paired t-test for BMC change within groups
[b] Unpaired t-test for difference in BMC change between groups.
